# Mitochondrial respiratory function in human platelets: Influence of sample preparation, assay buffer, and instrumental platform

**DOI:** 10.14814/phy2.70555

**Published:** 2025-09-30

**Authors:** Craig Porter, Lillie Treas, Oleksandra Pavliv, Daniel Sadler, Matthew Cotter, Shannon Rose, Elisabet Børsheim, Eva C. Diaz Fuentes

**Affiliations:** ^1^ Arkansas Children's Nutrition Center Little Rock Arkansas USA; ^2^ Arkansas Children's Research Institute Little Rock Arkansas USA; ^3^ University of Arkansas for Medical Sciences Little Rock Arkansas USA

**Keywords:** bioenergetics, mitochondria, platelets, respiration

## Abstract

Circulating blood cells such as platelets represent a readily available sample type to determine mitochondrial function in humans. Here, we set out to determine the influence of sample preparation, assay buffer composition, and instrumental platform on the respiratory function of platelets isolated from human blood. Approximately 50 mL of whole blood was collected from healthy adults (*n* = 16) following an overnight (>12 h) fast. Platelets were immediately isolated from whole blood by centrifugation for respirometry. Respiratory function was assayed in intact and permeabilized platelets using an Oxygraph‐2K (O2K) high‐resolution respirometer in either RPMI or MIR05 (containing 5 mM glucose, 1 mM pyruvate, and 2 mM glutamine), or the participant's own plasma. In addition, respiratory function was determined in intact platelets using a Seahorse Extracellular Flux analyzer (XFe96) in RPMI buffer containing 1 mM pyruvate, 2 mM glutamine, and variable glucose concentrations (5, 10, and 10 mM). In assays performed in an O2K, routine and ATP‐linked respiration were greater in cells assayed in RPMI compared to MIR05 (*p* < 0.001). However, compared to cells assayed in RPMI or MIR05, routine and ATP‐linked respiration were higher in intact platelets assayed in their own plasma (*p* < 0.001). In digitonin‐permeabilized platelets, state 3 respiration was greater when assayed in MIR05 compared to RPMI (*p* < 0.05). Across instrumental platforms, routine and leak respiration were lower in intact platelets assayed on an O2K versus an XFe96 (*p* < 0.05), whereas respiration available for ADP phosphorylation was greater in cells assayed on an O2K versus an XFe96 (*p* < 0.001), due to a diminished coupling response to oligomycin in cells assayed on the XFe96 (*p* < 0.001). Platelet respiratory function is influenced by assay buffer composition and instrumental platform. Consideration of these factors should be made by investigators planning to use platelet respiratory function as a readout of cellular energetics.

## 1 INTRODUCTION

Altered cellular energetics are implicated in the etiology of several chronic diseases (Wallace, 1999; Wallace, 2005), making the measurement of mitochondrial function an important readout for researchers interested in metabolic health and disease. In humans, access to tissue samples can be a barrier to making measurements of mitochondrial function. Circulating blood cells such as platelets represent an accessible cell type to determine mitochondrial function in humans. Initial interest in quantifying mitochondrial function in blood cells such as peripheral blood mononuclear cells (PBMCs) or platelets stemmed from the potential that altered bioenergetics of blood cells may also reflect altered mitochondrial function in specific organs (Braganza et al., 2019; Molina, 2017; Rose et al., 2019; Tyrrell et al., 2016), providing a minimally invasive “virtual” biopsy. In addition, there is growing interest in the role of blood cell energetics and immunometabolism in the etiology of numerous disease processes, making mitochondrial function of human PBMCs and platelets an important marker of disease progression and/or a readout for the efficacy of therapeutic interventions (Westerlund et al., 2018). Indeed, there has been a recent expansion of research efforts focused on characterizing bioenergetics of both PBMCs and platelets in various healthy and patient populations (Hoppel et al., 2021), where factors such as age, sex, and body mass index (BMI) have been shown to correlate with blood cell energetics (Sjövall et al., 2012). Furthermore, blood cell energetics are altered in numerous clinical populations, and in some, may predict morbidity and mortality (Sjövall et al., 2010; Sjövall et al., 2013).

Whole blood is primarily composed of red blood cells, leukocytes (monocytes, lymphocytes, and granulocytes), and platelets. In contrast to PBMCs, platelets represent a relatively homogenous cell population that can be readily isolated from blood (Vernerova et al., 2021). However, there is limited information on the influence of the instrumental platform used to assay respiratory function (O_2_ electrode approach in suspended cells versus a fluorometric approach in adherent cells), sample preparation, assay reagents, and respiration assay protocol on platelet energetics. Here, we set out to determine the influence of cell preparation, assay buffer composition, assay design, and instrumental platform on the measured respiratory function of platelets isolated from human blood.

## 2 METHODS

### 2.1 Participants

This study was approved by the Institutional Review Board at the University of Arkansas for Medical Sciences (IRB# 260236) and registered at clinicaltrials.gov (ClinicalTrials.gov Identifier: NCT04470505). Informed written consent was obtained from 16 young (25–35 years) healthy (BMI from ≥18.5 to ≤30 kg/m^2^) non‐smoking adults. Demographics for these participants are presented in Table 1. After screening, participants attended one study visit at Arkansas Children's Nutrition Center.

**TABLE 1:** Participant demographics.

Age (years)	29.8 ± 3.0
Male:Female Sex	7:9
Height (cm)	169.2 ± 10.5
Weight (kg)	66.5 ± 11.6
Body mass index (kg/m^2^)	23.1 ± 2.5
Systolic blood pressure (mmHg)	117 ± 11
Diastolic blood pressure (mmHg)	77 ± 7
Resting heart rate (bpm)	70 ± 12

### 2.2 Anthropometrics and vital signs

In the overnight‐fasted state, height and body mass (Seca 877; Seca GbmH & Co. KG, Hamburg, Germany) were recorded to the nearest 0.1 cm and 0.1 kg, respectively, and duplicate values were averaged. Blood pressure was measured in duplicate at 1‐min intervals on the right arm using an electronic vital sign monitor (CARESCPE™ VC150, Milwaukee, WI).

### 2.3 Blood sample collection

Approximately 50 mL of blood was drawn from the antecubital vein via venipuncture following an overnight fast. Briefly, a tourniquet was used to identify a suitable vein for venipuncture. Once a needle (18–20 gauge) was placed, the tourniquet was removed prior to blood collection. Approximately 2 mL of blood was then collected into a vacutainer and discarded. Thereafter, blood was collected into EDTA (ethylenediaminetetraacetic acid) coated vacutainers. EDTA vacutainers were gently inverted following the blood collection, kept at room temperature, and immediately transferred to the laboratory.

### 2.4 Blood processing for platelet isolation

Approximately 30 min post collection, platelets were isolated by centrifugation (using a Sorvall Legend X1 Centrifuge, ThermoFisher Scientific). EDTA vacutainers were centrifuged at 200×*g* for 10 min (acceleration 9, no brake) at room temperature. Platelet‐rich plasma was then recovered from the EDTA vacutainers into a 15 mL tube, leaving a 2–4 mm margin above packed red cells. Thereafter, 100 mM egtazic acid (EGTA, 10% by volume) was added to plasma to prevent platelet aggregation. This EGTA plasma was then centrifuged at 1000×*g* for 10 min (acceleration 6, brake 2). Platelet‐poor plasma was then recovered and stored on ice. The remaining platelet pellet was suspended in 4 mL of Dulbecco's phosphate‐buffered saline (DPBS) containing 10 mM EGTA. Following a final centrifugation at 1000×*g* for 5 min at room temperature (acceleration 6, brake 2), platelets were resuspended in 0.5 mL of DPBS containing 10 mM EGTA.

### 2.5 Platelet count

Platelet concentration (cells per volume) was determined spectrophotometrically, as described previously (Walkowiak et al., 1997). Briefly, 50 μL of suspended platelets were diluted in 450 μL of room temperature DPBS containing 10 mM EGTA. Blanks (200 μL of DPBS containing 10 mM EGTA) and samples (200 μL of platelets suspended in DPBS containing 10 mM EGTA) were pipetted into a flat‐bottom 96‐well microplate. The absorbance of blanks and platelet samples was read in a spectrophotometer (BioTek Epoch2, Winooski, VT) at 750 and 800 nm, and platelet number per mL was calculated (Equation 1)

(1) $$N=\left(\frac{6.23}{2.016-\mathrm{abs}\times 750/800}-3.09\right)\times 10\times 100$$

### 2.6 Respirometry

Analysis of mitochondrial respiration was performed on two widely used instrumental platforms. High‐resolution respirometry was performed on both intact and digitonin‐permeabilized platelets using three O2K respirometers (Oroboros Instruments, Innsbruck, Austria). Oxygen flux analysis was performed in intact platelets using a Seahorse XFe96 (Seahorse XFe9696, Agilent Technologies, Santa Clara, CA, USA).

#### 2.6.1 O2K instrumental set up

Three O2K respirometers were used simultaneously in this study, providing six 2 mL chambers for specific assays. Daily air calibrations were performed on the polygraphic oxygen sensor (POS) of each O2K chamber on the morning of each experiment. These calibrations were performed with a gas phase in the O2K chamber to allow equilibration of the respiration buffer with room air oxygen partial pressure. Either RPMI or MIR05 was used for air calibrations depending on the specific assay that would be performed in that chamber (see Tables S1 and S2 for details on the composition of RPMI and MIR05). Oxygen solubility factors of 0.89 and 0.92 were entered into the DatLab software (Oroboros Instruments, Innsbruck, Austria) for RPMI and MIR05, respectively. For the O2K chamber where plasma was used as a respiration buffer, background calibrations were performed in RPMI using an oxygen solubility factor of 0.89. Air calibrations were performed after about 45–60 min when the O_2_ slope in each chamber stabilized at ±1 pmol·s^−1^·mL^−1^, per the manufacturers operating procedures. A POS test was also performed each morning by turning off the stir bars within each O2K chamber for 30 s with the aim of observing a near instantaneous restoration in the chamber's O_2_ signal once stirring recommenced.

In addition to a daily POS test and air calibration, zero oxygen calibrations and instrumental background calibrations were performed using dithionite. Zero calibrations were performed periodically during the study period. Instrumental background calibrations were performed prior to commencing this study and any time POS was serviced. All instrumental background calibrations were performed in MIR05. For all O2K experiments, the temperature of the respiration buffer was maintained at 37°C. O_2_ concentration was maintained between ~100 and 190 μM in respiration buffers during all assays. Assay buffers were stirred rigorously throughout the experiments by a magnetic stir bar (750 rpm).

#### 2.6.2 O2K assays

Approximately 200 million platelets were resuspended in 2 mL of respiration buffer in each O2K chamber. The O_2_ concentration within the respiration buffer was recorded at 2–4 s intervals, from which O_2_ flux per million cells was calculated (DatLab, Oroboros Instruments, Innsbruck, Austria). Respiration was determined in both intact and digitonin‐permeabilized platelets in O2Ks. Respiration in intact platelets was assayed in both RPMI, MIR05, and the participant's own plasma (see Tables S1 and S2). Digitonin‐permeabilized platelets were assayed in both RPMI and MIR05.

#### 2.6.3 XFe96 instrumental set up

One XFe9696 instrument was used in this study. The XFe9696 was turned on the evening prior to experiments and the heater set to 37°C. An XF cartridge was also hydrated with 200 μL Agilent Seahorse XF Calibrant per well the day before experiments and kept at room temperature. On the day of experiments, the hydrated XF cartridge was placed in a 37°C non‐CO_2_ incubator, and an XF 96 PS plate was coated with poly‐d‐lysine as described previously (Rose et al., 2019).

#### 2.6.4 XFe96 assays

Ten million platelets (in a volume of 50 μL) were plated in each well of a poly‐D‐Lysine‐coated XF 96 PS (8 wells were used for each specific assay). Plates were then centrifuged at 1000×*g* for 2 min with no brake, then rotated 180 degrees and centrifuged again at 1000×*g* for 2 min with no brake. The plate was then kept in a non‐CO_2_ incubator at 37°C for 30 min. Thereafter, the volume of each well was adjusted to 180 μL by the addition of pre‐warmed RPMI assay media. During respiration assays, oxygen consumption rates (OCR) were determined over 3‐min increments. Three measures of routine (basal) OCR were made before the sequential addition of respiratory inhibitors and uncouplers (see below). Oxygen consumption rates from the XFe9696 were reported as pmol/min (per 10^7^ cells). In data presented for XFe9696 assays in Figures 6 and 7 all respiration values were converted to pmol/s/10^6^ cells to be consistent with the reporting of respiration from the O2K platform.

### 2.7 Respiration buffers

Respiration experiments were either performed in RPMI assay media or MIR05 buffer. Details regarding the composition of these RPMI or MIR05 buffers can be found in Tables S1 and S2, respectively. The sources of chemicals and reagents used to make these two buffers can be found in Table S3. Further, comparisons of the pH, osmolality, ionic strength, and oxygen solubility of RPMI and MIR05 used in the present study are presented in Table S3.

Assays were performed on O2K instruments using RPMI (pH 7.4) MIR05 (pH 7.4), or in the participants own plasma. RPMI and MIR05 were supplemented with glucose (5 mM), pyruvate (1 mM) and glutamine (2 mM) for all O2K assays. Assays performed on the XFe96 were performed using RPMI (pH 7.4) containing glucose (5 mM), pyruvate (1 mM), and glutamine (2 mM). For some assays performed on the XFe96, RPMI assay media were supplemented with either 10 mM or 20 mM glucose.

### 2.8 Respiration assays

**FIGURE 1:**
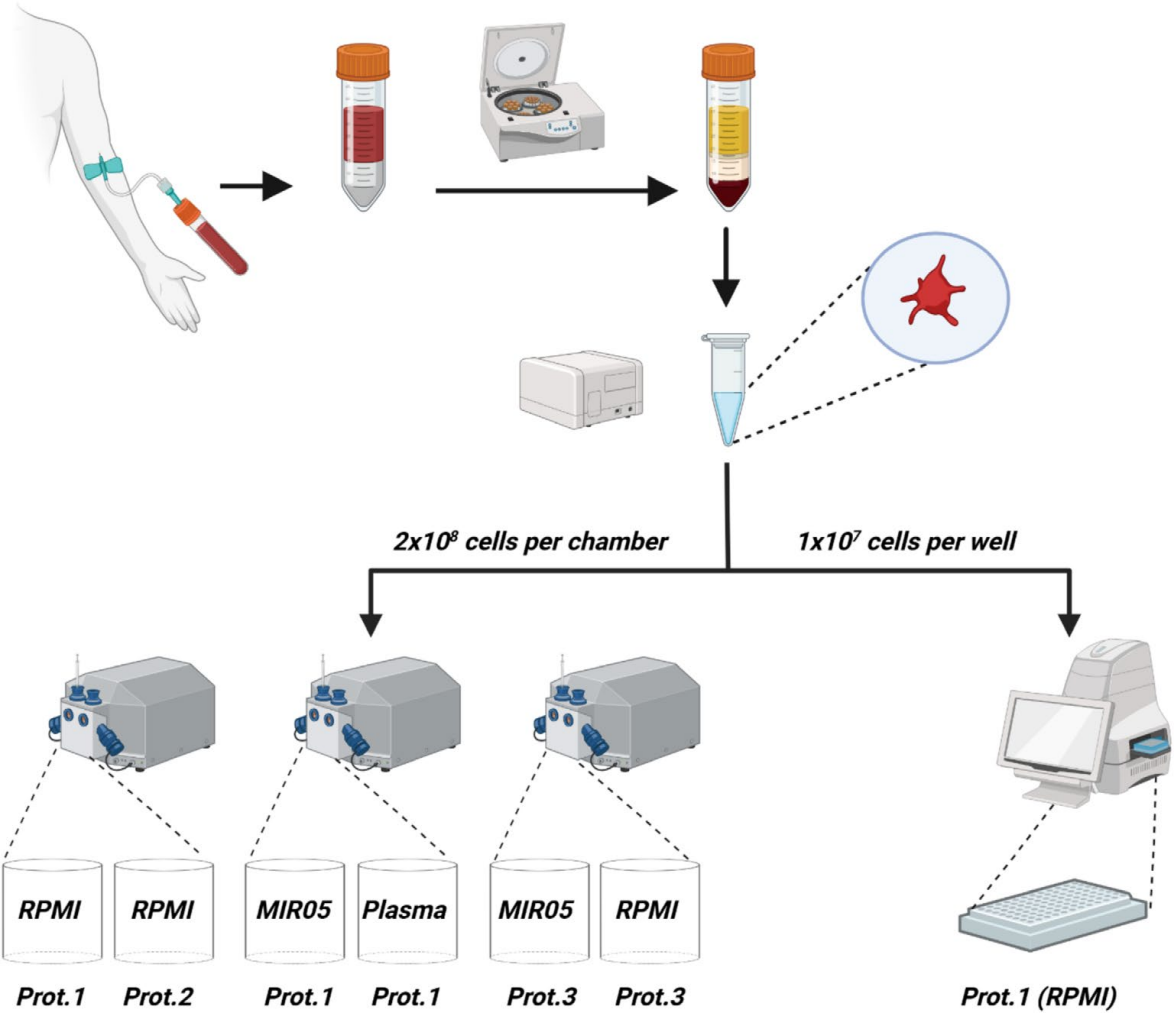
Schematic overview of study design. Platelets were isolated from approximately 50 mL of whole blood by gradient centrifugation. Platelets were then either plated (10 million per well) in a 96‐well plate for Seahorse XFe9696 or loaded (200 million per chamber) into the chamber of an O2K respirometer for oxygen consumption analysis. Depending on the total yield of platelets isolated from each participant, up to 3 different respiratory protocols were employed over the two instrumental platforms (i.e., the XFe9696 and O2K). For assays performed on the XFe9696, respiration was measured in intact platelets using RPMI containing different glucose concentrations. For assays performed on O2Ks, respiration was measured in intact platelets suspended in either RPMI, MIR05, or the participant's own platelet poor plasma (O2Ks 1 and 2). Respiration was also measured in digitonin‐permeabilized platelets suspended in either RPMI or MIR05 (O2K 3). Further details on experimental buffers and assay protocols can be found in the Tables S1–S9). Figure 1 was produced in BioRender.

Figure 1 provides an overview of how platelets from each participant were used for respirometry analysis. Three different respiration protocols were used to determine mitochondrial respiratory capacity in various coupling states in both intact and permeabilized platelets. For intact platelets, a widely used mitochondrial stress test was performed where, after recording routine respiration, oligomycin (1 μM) and then carbonyl cyanide p‐(tri‐fluromethoxy)phenyl‐hydrazone (FCCP, 0.5 μM additions to a final concentration of 1.5 μM) were used to determine mitochondrial respiration in the leak and uncoupled states, respectively. Thereafter, the Complex I inhibitor rotenone (1 μM) and Complex III inhibitor antimycin A (5 μM) were added to each well, and non‐mitochondrial respiration was determined (Protocol 1, see Table S5 for further details). For experiments performed on O2Ks, N,N′, N′‐tetramethyl‐1,4‐benzenediamine dihydrochloride (TMPD, 0.5 mM) and ascorbate (2 mM) were also added to chambers following the addition of antimycin A in order to determine Complex IV‐driven respiration (see Table S6 for further details). To determine the impact of oligomycin on FCCP‐driven uncoupled respiration in intact platelets, a modified version of the respiration protocol described above was used, where the addition of oligomycin was omitted (Protocol 2). This assay was performed on both instrumental platforms (Tables S7 and S8).

A third protocol was used to assay respiratory function of permeabilized platelets (see Supplemental Table S9). Briefly, after recording routine respiration in intact cells, platelets were then permeabilized with digitonin (2 μg/1×10^8^ cells). Thereafter, 5 mM ADP followed by 2 mM malate and 10 mM glutamate were added to O2K chambers to determine state 3 respiration supported by Complex I. Succinate (50 mM) was then added to determine state 3 respiration supported by Complex I and Complex II. Then, respiration in the leak was determined after the addition of the ATP synthase inhibitor oligomycin (1 μM). To uncouple respiration, FCCP was titrated into the O2K chamber in 0.5 μM increments to a final concentration of 1.5 μM. Thereafter, rotenone (0.5 μM) and antimycin A (5 μM) were added to each well and non‐mitochondrial respiration determined. Finally, TMPD (0.5 mM) and ascorbate (2 mM) were added to O2K chambers to assay Complex IV driven respiration (Protocol 3).

Finally, for assays performed on the XFe96, Protocol 1 was repeated in RPMI supplemented with 10 mM to 20 mM glucose. In all instances, RPMI contained 1 mM pyruvate and 2 mM glutamine.

### 2.9 Protein and citrate synthase activity assays

Citrate synthase (CS) activity was measured as a surrogate of mitochondrial protein abundance using a commercially available kit (Sigma‐Aldrich, Burlington, MA, USA). Platelet pellets were lysed in 1% Triton X‐100 containing 150 mM NaCl and 50 mM Tris (pH 8.0). CS activity was determined in lysates in a 100 mM phosphate buffer (pH 7.1) containing 10 mM 5,5′‐dithiobis‐(2‐nitrobenzoic acid) (DTNB) and 30 mM acetyl‐CoA. After the addition of 10 mM oxaloacetate, free coenzyme A produced from the condensation of acetyl‐CoA and oxaloacetate was bound to DTNB. The resulting change in light absorbance was measured spectrophotometrically at 37°C and 412 nm (BioTek Instruments, Winooski, VT, USA), and was used to determine CS activity (nmol·s^−1^·mL^−1^). CS activity was then normalized to protein content determined by the Bradford assay, as per manufacturer instructions (Sigma‐Aldrich, Burlington, MA, USA).

### 2.10 Data analysis

DatLab (Oroboros Instruments, Innsbruck, Austria) and Wave files (Seahorse Wave Software, Agilent Technologies, Santa Clara, CA, USA) for each assay were exported to a Microsoft Excel file. All respiratory fluxes were corrected for residual (non‐mitochondrial) respiration, that is, respiration following the titration of rotenone and antimycin A was subtracted from all other respiration rates. Coupling efficiency was derived from the coupling control response to oligomycin. Specifically, for Protocols 1 and 2, the coupling efficiency of intact platelets was calculated as 1‐(leak/routine). For Protocol 3, the coupling efficiency of permeabilized platelets was calculated as 1‐(State 4_O_/State 3_I+II_). In all protocols, the respiratory capacity available for ADP phosphorylation represents oligomycin‐sensitive respiration.

All values are presented as group means ± standard deviations unless stated otherwise. Differences between groups were assessed using a one‐way analysis of variance or *t*‐test where appropriate. Statistical analyses were performed using GraphPad Prism Version & (La Jolla, CA).

## 3 RESULTS

Participant demographics are shown in Table 1. Sixteen healthy adults provided blood samples for the platelet respirometry analysis described herein. Participants included both males (*n* = 7) and females (*n* = 9). All participants had a healthy BMI and were normotensive.

### 3.1 Assay buffer composition impacts respiration in intact platelets

**FIGURE 2:**
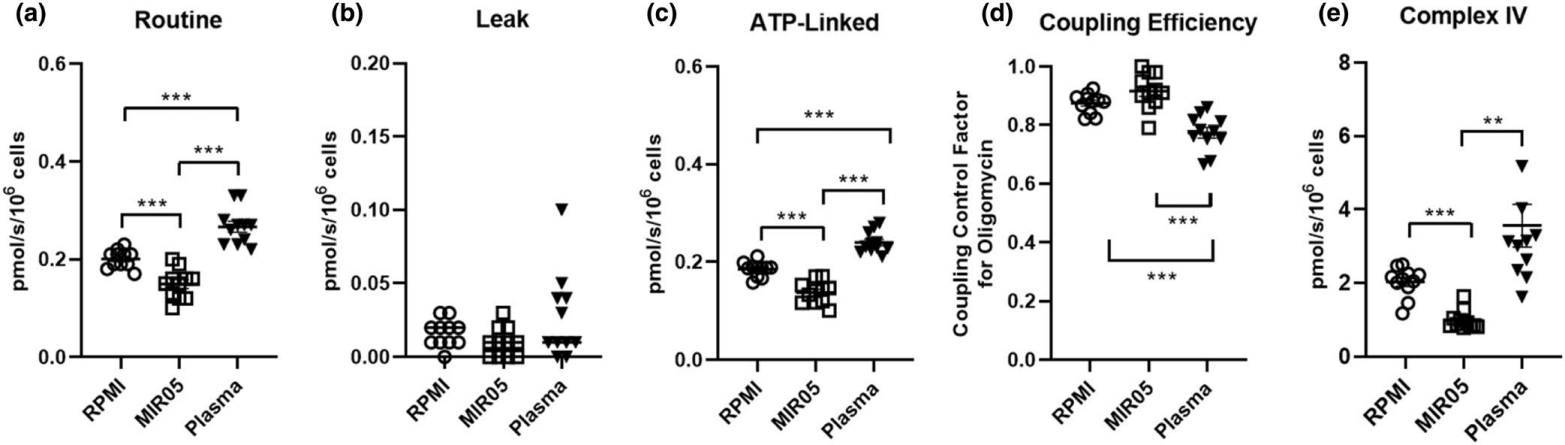
Impact of buffer composition on respiration in intact platelets. Respiration was measured concurrently using this protocol on three separate O2K instruments where isolated platelets were resuspended in either RPMI (supplemented with glucose [5 mM], pyruvate [1 mM] and glutamine [2 mM]), MIR05 supplemented with glucose (5 mM), pyruvate (1 mM), and glutamine (2 mM)), or the participant's own platelet poor plasma. (Panel a and b) show routine and leak respiration for the 3 experimental conditions. (Panel c) shows ATP‐linked respiration (routine–oligomycin insensitive leak respiration). (Panel d) shows the coupling efficiency for the 3 experimental conditions expressed as the coupling control factor for oligomycin (i.e., 1–coupling control ratio for oligomycin). (Panel e) shows Complex IV‐driven respiration efficiency for the 3 experimental conditions. Individual values for each participant (*n* = 11) are plotted, as are group means ± standard deviations. **p < 0.01, ***p < 0.001.

Respiration measured in intact platelets suspended in RPMI or MIR05 (both supplemented with glucose [5 mM], pyruvate [1 mM], and glutamine [2 mM]), or the participant's own platelet poor plasma is shown in Figure 2. Panels a and b show routine and oligomycin insensitive leak respiration. Panel c shows respiratory capacity available for ADP phosphorylation (routine–oligomycin insensitive respiration). Routine respiration and respiration available for ADP phosphorylation were all higher in intact platelets assayed in RPMI compared to MIR05 (*p* < 0.001). Compared to both RPMI and MIR05, routine respiration and respiration available for ADP phosphorylation were higher in intact platelets assayed in platelet poor plasma (*p* < 0.001). Leak respiration was not significantly different across the three experimental groups. Panel d shows the coupling efficiency expressed as the coupling control factor for oligomycin (i.e., 1–coupling control ratio for oligomycin). Coupling efficiency of intact platelets assayed in RPMI or MIR05 was comparable; however, measured coupling efficiency was significantly lower in intact platelets assayed in platelet poor plasma compared to cells assayed in either RPMI or MIR05 (*p* < 0.001). Panel e shows Complex IV‐driven respiration, which was greater in intact platelets assayed in RPMI (*p* < 0.001) and platelet poor plasma (*p* < 0.01) when compared to MIR05.

### 3.2 Assay buffer composition impacts respiration in permeabilized platelets

**FIGURE 3:**
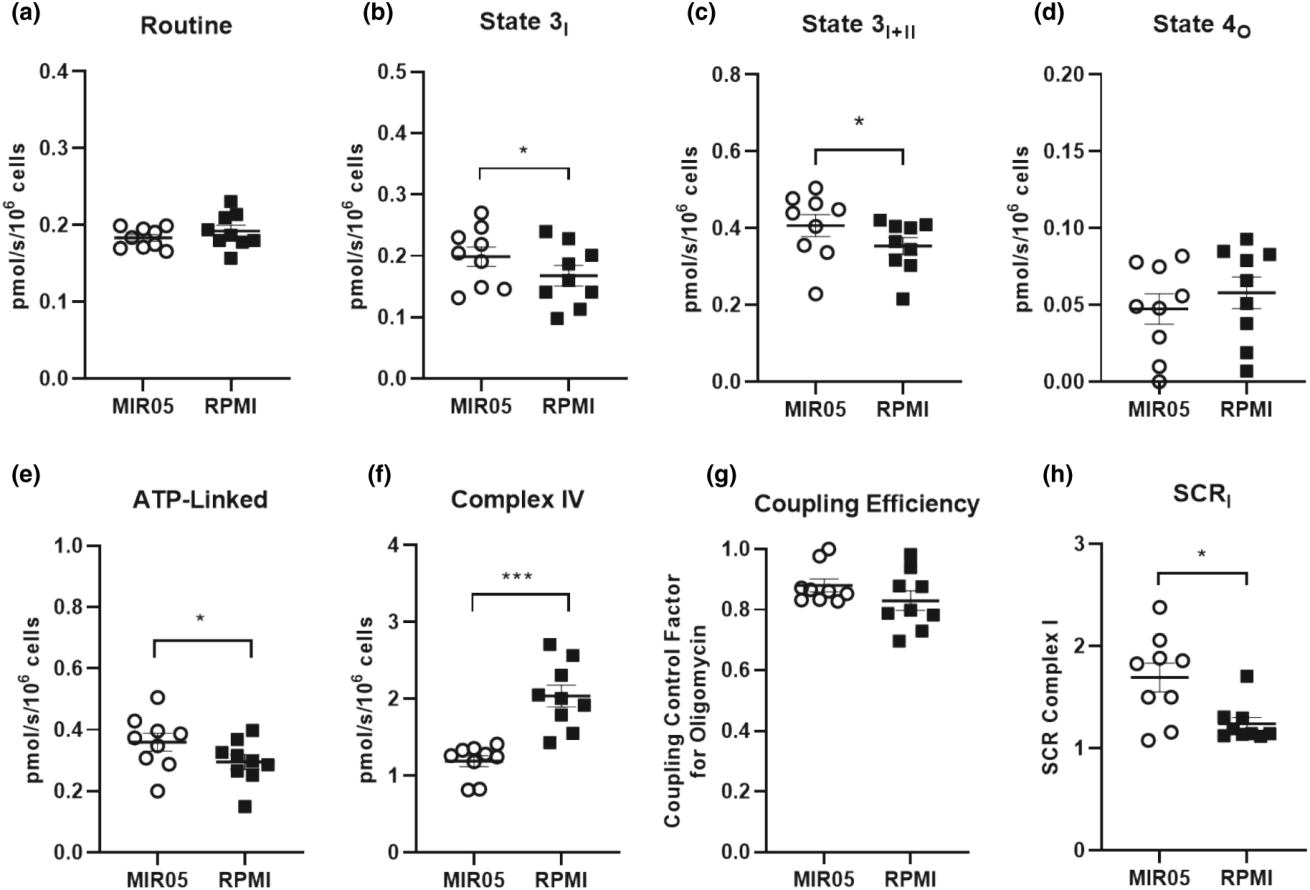
Impact of buffer composition on respiration in permeabilized platelets. (Panel a) depicts the protocol used to assay mitochondrial respiratory capacity in permeabilized platelets. Respiration was measured concurrently using this protocol on a single O2K instrument where isolated platelets were resuspended in either RPMI (supplemented with glucose (5 mM), pyruvate (1 mM), and glutamine (2 mM)) or MIR05 supplemented with glucose (5 mM), pyruvate (1 mM), and glutamine (2 mM)). (Panels b–d) show routine, complex I supported state 3, and complex I+II supported state 3 respiration for the 3 experimental conditions, respectively. (Panel e) shows ATP‐linked respiration (routine–oligomycin insensitive leak respiration). (Panel f) shows complex IV respiration supported by TPMD and ascorbate. (Panel g) shows the coupling efficiency for the 3 experimental conditions expressed as the coupling control factor for oligomycin (i.e., 1–coupling control ratio for oligomycin). (Panel h) shows the substrate control ratio for Complex I, calculated as the ratio of respiration before and after addition of malate and glutamate. Individual values for each participant (*n* = 9) are plotted, as are group means ± standard deviations. *p < 0.05, ***p < 0.001.

Respiration was measured in digitonin‐permeabilized platelets suspended in either MIR05 or RPMI (both supplemented with glucose (5 mM), pyruvate (1 mM), and glutamine (2 mM)) (Figure 3). Panel a shows routine respiration, which was comparable in intact platelets assayed in either RPMI or MIR05. Panels b and c show state 3 respiration supported by complex I and complex I + II, respectively, both of which were significantly higher in permeabilized platelets assayed in MIR05 versus RPMI (*p* < 0.05). Oligomycin‐insensitive state 4_O_ respiration was not different between permeabilized platelets assayed in MIR05 versus RPMI (Panel d). Panel e shows respiration available for ADP phosphorylation (routine–oligomycin insensitive leak respiration), which was significantly higher in permeabilized platelets assayed in MIR05 versus RPMI (*p* < 0.05). Panel f shows complex IV respiration supported by TPMD and ascorbate, which was significantly lower in permeabilized platelets assayed in MIR05 versus RPMI (*p* < 0.001). Coupling efficiency expressed as the coupling control factor for oligomycin (i.e., 1–coupling control ratio for oligomycin) was not different in permeabilized platelets assayed in MIR05 versus RPMI (Panel g). Panel h shows the substrate control ratio for Complex I (SCR_I_), calculated as the ratio of respiration before and after the addition of malate and glutamate. The (SCR_I_) was significantly higher in permeabilized platelets assayed in MIR05 versus RPMI (*p* < 0.05).

### 3.3 Oligomycin influences protonophore driven respiration

**FIGURE 4:**
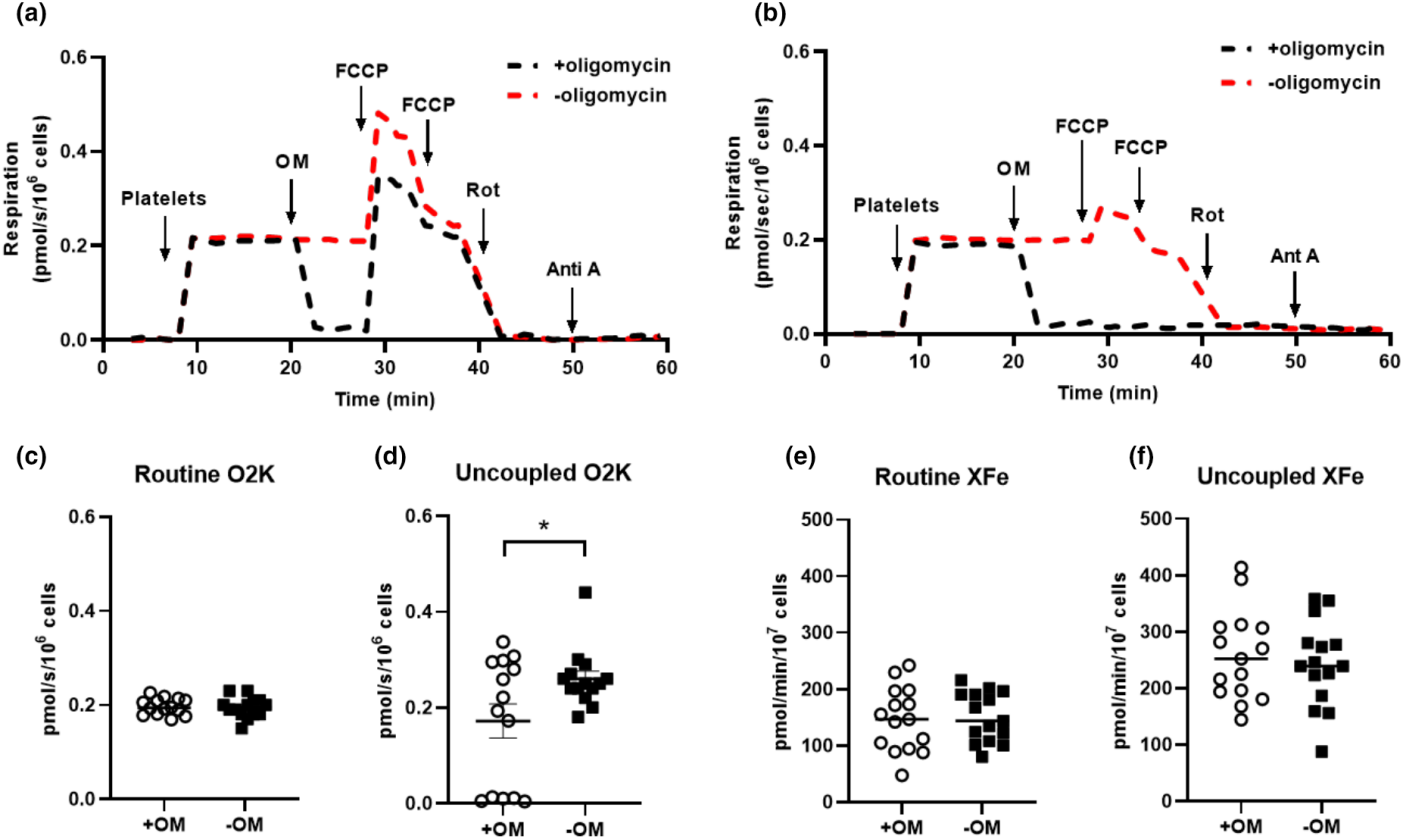
The impact of oligomycin on protonophore driven respiration. (Panel a and b) depict paired measurements of respiration in platelets isolated from the same individual assayed concurrently on a single O2K instrument. For both assays, intact platelets were resuspended in RPMI (supplemented with glucose [5 mM]), pyruvate [1 mM], and glutamine [2 mM]). In one O2K chamber, oligomycin (1 μM) was added to determine respiration linked to proton leak, prior to the titration of FCCP (0.5 μM additions) to assay uncoupled respiratory capacity. In the other O2K chamber, FCCP titration was performed in the absence of oligomycin. (Panels a and b) depict to different samples, one where platelets responded to FCCP in the presence of oligomycin (Panel a), and one where titration of FCCP failed to ellicit a respiratory response in the presence of oligomycin (Panel b). (Panels c and d) show routine and uncoupled respiration for the two assays conditions. Similarly, (Panels e and f) show routine and uncoupled respiration for the assay conditions for intact platelets analyzed on the XFe9696. Individual values for each participant (*n* = 14 for O2K experiments in Panels c and d), (*n* = 16 for XFe96 experiments in Panels e and f) are plotted as group means ± standard deviations. *p < 0.01.

Panel a and b of Figure 4 both depict representative measurements of respiration in intact platelets isolated from the same individual assayed concurrently on the same O2K instrument. For both assays, platelets were resuspended in RPMI (containing glucose (5 mM), pyruvate (1 mM), and glutamine (2 mM)). In one chamber, oligomycin (1 μM) was added prior to the titration (0.5 μM of FCCP). In the other chamber, FCCP titration was performed in the absence of oligomycin. Panel a depicts an experiment where, although a respiratory response that was greater than routine respiration could be measured following the addition of FCCP, the measured respiratory capacity of the electron transfer system of intact platelets was diminished in the presence of oligomycin. Panel b shows that some intact platelets (~35% of samples in the current study) did not respond to FCCP titration in the presence of oligomycin. This was the case both for intact and permeabilized platelets, respectively (data from permeabilized platelets are shown in Figure S1). Panels c and d show routine and uncoupled respiration for the two assay conditions when assayed on an O2K, where FCCP‐induced uncoupled respiration was significantly lower in intact platelets when assayed in the presence versus absence of oligomycin (*p* < 0.05). Routine respiration assayed in intact platelets on an XFe96 is shown in Panel e. In contrast to the data shown in Panel d, FCCP‐induced uncoupled respiration was not impacted by oligomycin when assayed on an XFe96 (Panel f).

### 3.4 Mitochondrial respiratory capacity and coupling control in intact versus permeabilized platelets

**FIGURE 5:**
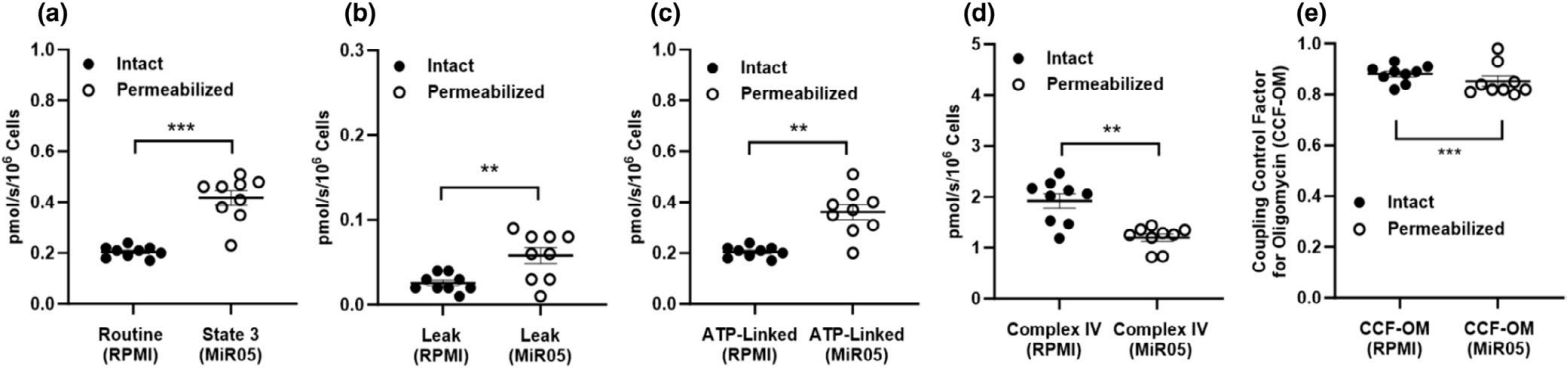
Mitochondrial respiratory capacity and coupling control in intact versus permeabilized platelets. (Panel a) shows routine respiration in intact versus state 3 (supported by Complex I and II) in permeabilized platelets. (Panel b) shows oligomycin insensitive leak respiration in intact versus permeabilized platelets. (Panel c) shows oligomycin sensitive (i.e., respiration linked to ATP production) respiration in intact versus permeabilized platelets. (Panel d) shows TMPD and ascorbate complex IV‐driven respiration in intact versus permeabilized platelets. (Panel e) shows the coupling control factor for oligomycin (CCF‐OM) calculated as 1–coupling control ratio for oligomycin in intact versus permeabilized platelets. Individual values for each participant (*n* = 9) are plotted, as are group means ± standard deviations. The highest respiratory fluxes for each cell preparation (intact vs. permeabilized) were used, thus intact cells assayed in RPMI were compared to permeabilized cells assayed in MIR05. **p < 0.01, ***p < 0.001.

Figure 5 depicts respiratory rates and coupling states assayed in either intact or permeabilized platelets. Panel a compares routine respiration assayed in intact platelets versus state 3 respiration (supported by Complex I and II) assayed in permeabilized platelets. State 3 respiration in permeabilized platelets was ~2‐fold greater than routine respiration assayed in intact platelets (*p* < 0.001). Panel b shows oligomycin‐insensitive leak respiration, which was also greater when assayed in permeabilized platelets versus intact platelets (*p* < 0.05). Panel c shows oligomycin‐sensitive respiration (i.e., respiration available for ADP phosphorylation) which was ~2‐fold greater when assayed in permeabilized platelets versus intact platelets (*p* < 0.001). TMPD and ascorbate‐driven Complex IV‐driven respiration in intact versus permeabilized platelets is shown in Panel d. In contrast, Complex IV‐driven respiration was greater when assayed in intact versus permeabilized platelets (*p* < 0.001). Panel e shows the coupling control factor for oligomycin (i.e., 1–coupling control ratio for oligomycin) assayed in intact versus permeabilized platelets. The coupling control factor for oligomycin was greater when assayed in intact platelets compared to permeabilized platelets (*p* < 0.001).

### 3.5 The impact of buffer glucose concentration on respiration in intact platelets

**FIGURE 6:**
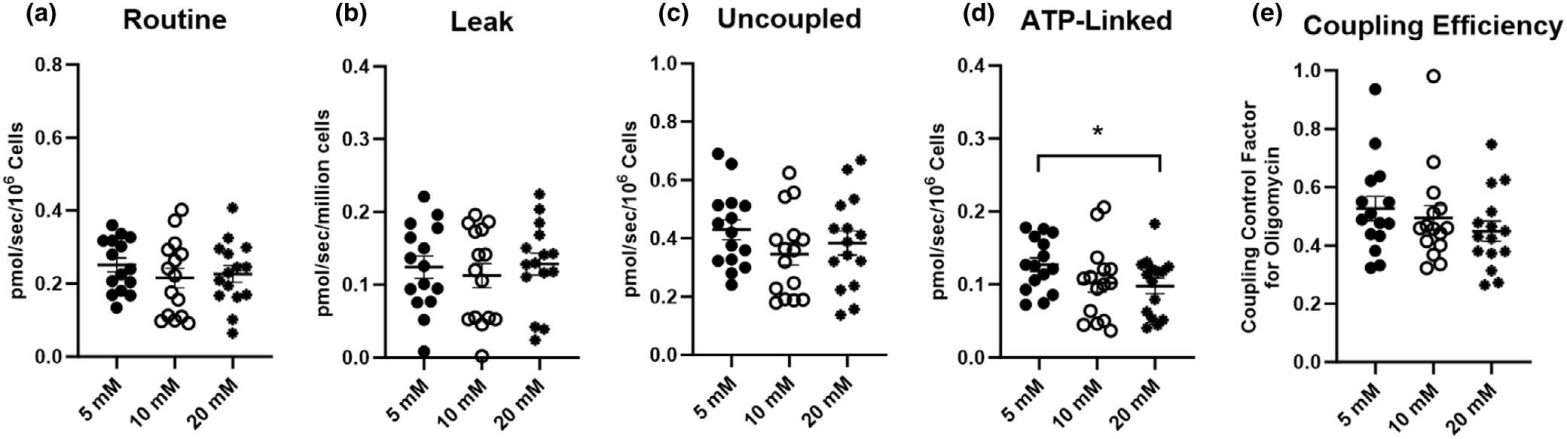
The impact of buffer glucose concentration on respiration in intact platelets. Routine, leak, and uncoupled respiration assayed in intact platelets on a Seahorse XFe9696 are shown in (Panel a, b, and c), respectively. Platelets from the same participant (1 × 10^7^ cells per well) were assayed in RPMI containing 1 mM pyruvate, 2 mM glutamine, and either 5, 10, or 20 mM glucose. (Panel d) shows oligomycin‐sensitive (i.e., respiration linked to ATP production) in intact platelets assayed at different glucose concentrations. (Panel e) shows the coupling control factor for oligomycin (i.e., 1–coupling control ratio for oligomycin) in intact platelets assayed at different glucose concentrations. Oxygen consumption rates from the XFe9696 were reported as pmol/min (per 10^7^ cells) and were converted to pmols/s/10^6^ cells to be consistent with the reporting of respiration across instrumental platforms. Individual values for each participant (*n* = 15) are plotted, as are group means ± standard deviations. *p < 0.05.

Routine, leak, and uncoupled respiration assayed in intact platelets on an XFe96 are shown in Panels a, b, and c of Figure 6, respectively. Platelets from the same participant (1 × 10^7^ cells per well) were assayed in RPMI containing 1 mM pyruvate, 2 mM glutamine, and either 5, 10, or 20 mM glucose. The glucose concentration of RPMI did not impact routine, leak, or uncoupled respiration in intact platelets. Panel d shows oligomycin‐sensitive respiration (i.e., respiration available for ADP phosphorylation) in intact platelets assayed at different glucose concentrations, where respiration linked to ATP production was lower in intact platelets assayed at 20 mM glucose compared to those assayed at 5 mM glucose (*p* < 0.01). Panel e shows that the coupling control factor for oligomycin (i.e., 1–coupling control ratio for oligomycin) assayed in intact platelets was not altered by glucose concentration.

### 3.6 Mitochondrial respiratory capacity and coupling control in platelets assayed on different instrumental platforms

**FIGURE 7:**
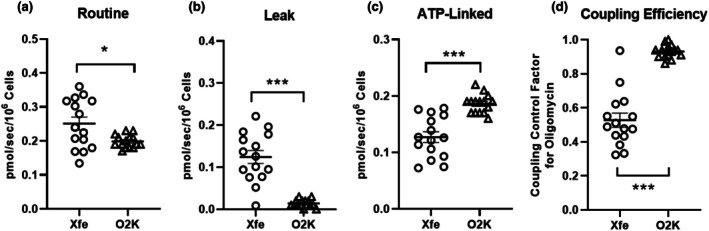
Mitochondrial respiratory capacity and coupling control in platelets assayed on different instrumental platforms. Routine, leak, and oligomycin sensitive (i.e., respiration linked to ATP production) are shown in (Panels a–c), respectively. All assays were performed in RPMI containing 5 mM glucose, 1 mM pyruvate, and 2 mM glutamine. 10 × 10^6^ cells per well were used for Seahorse XFe9696 assays and 200 × 10^6^ cells per mL for O2K assays. (Panel d) shows the coupling control factor for oligomycin (i.e., 1–coupling control ratio for oligomycin) in intact platelets from the same participant assayed on an XFe96 and O2K. Oxygen consumption rates from the XFe9696 are calculated as pmol/min (per 10^7^ cells) and were converted to pmols/s/10^6^ cells to be consistent with the O2K platform. Individual values for each participant (*n* = 15) are plotted as group means ± standard deviations. *p < 0.05, ***p < 0.001.

Routine, leak, and oligomycin sensitive (i.e., respiration available for ADP phosphorylation) assayed in intact platelets are shown in Panels a–c of Figure 7. All assays were performed in RPMI containing 5 mM glucose, 1 mM pyruvate, and 2 mM glutamine. 1 × 10^7^ cells per well were used for XFe96 assays and 2 × 10^8^ cells per mL for O2K assays. When expressed as respiration per second per million cells, routine respiration was significantly greater in intact platelets assayed on an XFe9696 versus O2K (*p* < 0.05). Similarly, leak respiration was also significantly greater in intact platelets assayed on an XFe96 versus O2K (*p* < 0.001). In contrast, respiratory capacity available for ADP phosphorylation was lower in intact platelets assayed on an XFe96 compared to O2K (*p* < 0.001). Panel d shows the coupling control factor for oligomycin (i.e., 1–coupling control ratio for oligomycin), which was lower in intact platelets assayed on an XFe96 compared to O2K (*p* < 0.001). We also assessed the coefficient of variance for technical replicates assayed on both platforms. Routine respiration was measured in the same buffer in three O2K chambers (on three different O2K instruments) in intact platelets isolated from *n* = 13 participants (Figure S1). The coefficient of variance for these three technical replicates ranged from 1.1% to 16% or 6.0 ± 5.2% on average (mean ± SD). Routine respiration was also assayed in *n* =5–6 replicates of intact platelets isolated on the same XFe96 plate from *n* = 15 participants. The coefficient of variance for these 5–6 technical replicates ranged from 7.7% to 57.1% or 29.4 ± 14.1% on average (mean ± SD).

### 3.7 Platelet respiratory capacity is not correlated to cellular protein or mitochondrial enzyme activity

Cell respiration can be influenced by both the quality and quantity of mitochondria. To account for differences in mitochondrial quantity, the concentration of specific mitochondrial proteins or the activity of specific mitochondrial enzymes can reflect differences in mitochondrial protein abundance. Various markers of mitochondrial protein content are often used to normalize respiratory data in an attempt to account for potential differences in mitochondrial protein content across samples. Here, we found that while there was a relationship between cell total protein concentration and the activity of the TCA cycle enzyme citrate synthase, there was no apparent relationship between routine respiration in intact platelets and either total protein concentration, citrate synthase activity, or the Cytochrome C Oxidase activity (Figure S3). Accordingly, cell respiration data were not further normalized to any of these three markers.

## 4 DISCUSSION

There is growing interest in the influence of sample processing, assay buffer and design, and instrumental platform on the analysis of platelet mitochondrial respiratory function. In the current study, we determined the influence of cell preparation (intact vs. permeabilized), assay buffer (RPMI vs. MIR05), assay design, and instrumental platform on the respiratory function of platelets isolated from the blood of healthy humans. We found that in intact platelets, respiratory capacity was greater when assayed in RPMI compared to MIR05, whereas the coupling efficiency of intact platelets was comparable when assayed in either RPMI or MIR05. In contrast, in permeabilized platelets, ADP‐driven state 3 respiration was greater in cells assayed in MIR05 compared to RPMI. Our results also suggest that oligomycin may diminish protonophore induced uncoupled respiration, meaning care should be taken when interpreting measures of electron transfer system capacity made in the presence of oligomycin. Respirometry can be readily performed in platelets on both O2K and XFe96 platforms. However, we found important incongruences in several readouts of respiratory function when comparing the two platforms.

A key finding of the current study was that assay buffer composition influenced respiratory capacity in platelets specific to sample preparation. In intact platelets, RPMI supported greater routine respiration and respiration available for ADP phosphorylation when compared to MIR05, whereas in permeabilized platelets, MIR05 supported greater routine respiration and respiration available for ADP phosphorylation when compared to RPMI. This is perhaps not surprising given the composition of the two buffers and known effects of these parameters on the kinetics of numerous reactions involved in bioenergetics (Bradshaw & Pfeiffer, 2006; Glancy et al., 2013; Golding et al., 1995; Shabalina et al., 2010). Indeed, RPMI's greater ionic strength and lower osmolality make it well suited to supporting intact cells. In contrast, MIR05 is designed to support the determination of respiratory function of mitochondrion in permeabilized tissue and isolated organelles, where its composition supports more optimal intracellular buffering relative to RPMI. Further, others have noted that relative to RPMI, MiR05 can result in the aggregation of intact PBMCs (Bager Christensen et al., 2024). While we did not determine the impact of RPMI and MiR05 on platelet aggregation in the current study, this may be a potential explanation for the observed lower respiration in intact platelets assayed in MiR05 versus RPMI.

In addition to studying the impact of RPMI and MIR05 on platelet respiration, we also determined the respiratory function of intact platelets resuspended in their own platelet‐poor plasma. The rationale for this was that while assaying cells suspended in specific respiration buffers allows for control over the extracellular or intracellular environments, it is somewhat removed from the physiological state that cells are exposed to in vivo. This is particularly true of platelets, where concentrations of metabolites, hormones, and cytokines in blood likely exert control of platelet respiratory function in vivo. Accordingly, assaying platelets in their own plasma may offer insight into the physiology of the cells when exposed to the metabolic and hormonal milieu present in whole blood. Intriguingly, we found that respiratory capacity in the routine state, as well as respiration available for ADP phosphorylation, was both greater in intact platelets assayed in their own plasma relative to cells assayed in either RPMI or MIR05. Interestingly, despite greater cell‐specific‐linked respiration, coupling control in response to oligomycin was diminished in platelets assayed in their own plasma. This diminished response to oligomycin may have been due to the protein or fatty acid content of plasma interfering with oligomycin binding to the F_0_ unit of ATP synthetase. Alternatively, factors within plasma that are absent in RPMI/MIR05 may promote proton leak. Indeed, fatty acid concentrations are known to control inner membrane proton leak in several cell types. To directly study the impact of substrate concentration on platelet respiratory function, we assayed routine, leak, and uncoupled respiration in intact platelets on an XFe96. Platelets from the same donor were assayed in RPMI containing 1 mM pyruvate, 2 mM glutamine, and one of three glucose concentrations (either 5, 10, or 20 mM). Interestingly, glucose concentration had no impact on platelets respiration, with the exception of respiration linked to ADP phosphorylation, which was lower in intact platelets assayed at 20 mM glucose compared to those assayed at 5 mM glucose. Overall, this suggests that glucose concentrations do not significantly alter platelet respiratory capacity.

The use of the macrolide oligomycin is widespread in respirometry experimentation owing to its ability to inhibit the *F*_0_ unit of ATP synthetase, thereby inhibiting respiration that is linked to ATP production. This allows the differentiation of respiration linked to proton leak and respiration available for ADP phosphorylation (i.e., respiration linked to ATP production). After the addition of oligomycin, many assays also add an uncoupler such as FCCP to then assay the respiratory capacity of the electron transfer system. However, it has been reported that the respiratory capacity of the electron transfer system assayed in the presence or absence of oligomycin may differ (Ruas et al., 2016). In intact human platelets, we also found that protonophore‐stimulated respiration was diminished when measured in the presence of oligomycin. In our experiments, 1 μM oligomycin was added to O2K chambers. This concentration of oligomycin resulted in the absence of a response to FCCP in ~one‐third of all samples. Others have reported that oligomycin similarly inhibits protonophore‐induced respiratory capacity at a concentration of 2.5 μM, but that reducing this concentration to 0.5 μM allowed for a robust response to the uncoupler (Doerrier et al., 2018; Sumbalová et al., 2018). Thus, it would appear that when measuring protonophore‐induced respiratory capacity in intact human platelets, optimization of oligomycin concentration is necessary.

Respiratory function can be assayed in intact cells or permeabilized cells. Typically, the detergent digitonin is used to permeabilize cells, allowing respiratory substrates and inhibitors that may not readily cross cell membranes to be used to interrogate respiratory function. Accordingly, respiratory function assayed in intact versus permeabilized cells is fundamentally different in terms of the condition of the cells being assayed and the substrates being used to drive respiration. Further, as we report here, assay buffer composition can also impact respiratory function in cells, at least in platelets–where RPMI appears better suited for supporting respiration in intact platelets, while MIR05 better supports respiration in digitonin permeabilized platelets. Using oligomycin sensitive respiration as a proxy of capacity for ATP production (or more precisely respiratory capacity available for ADP phosphorylation), we found that capacity for respiration available for ADP phosphorylation was approximately 2‐fold greater when measured in permeabilized versus intact platelets. This indicates that substrate and/or ADP availability limits measured capacity for respiration linked to ATP production in intact cells. While measured capacity for respiration linked to ATP was greater per cell in permeabilized versus intact platelets, so too was oligomycin sensitive respiration. Indeed, coupling control in response to oligomycin was lower in permeabilized versus intact platelets, demonstrating that while permeabilization allows for the measurement of greater respiratory capacity for both ADP phosphorylation and proton leak, permeabilization also appears to result in altered mitochondrial coupling control.

The O2K (Oroboros Instruments) and the Seahorse XFe96 (Agilent Technologies) are two widely used platforms to assay mitochondrial respiratory function in cells. Important differences in these two platforms have been discussed in detail elsewhere (Perry et al., 2013; Schmidt et al., 2021). Briefly, the O2K and XFe96 primarily differ in terms of detection method, where the O2K employs a Clark electrode based potentiometric method to determine respiration (Gnaiger, 2008) whereas the XFe96 employs a fluorometric approach to assay respiration (O'Donovan et al., 2005; Schmidt et al., 2021; Wilson et al., 1996; Zhdanov et al., 2010), likely leveraging metalporhyrin complexes (Gerencser et al., 2009). Another important difference in these two approaches is that cells are suspended for O2K assays (typically in between 0.5 and 2 mL of respiration buffer), while adherent cells are assayed by the XFe96. In our study, we found that when expressed as respiration per second per million cells‐routine respiration was significantly greater in intact platelets assayed on an XFe96 versus O2K. This is in agreement with a previous study that reported higher routine respiration in cells assayed on the XFe96 versus O2K (Jedlička et al., 2021). However, these authors (Jedlička et al., 2021) also reported a correlation between routine respiration cells assayed on the XFe9696 versus O2K, which was not the case in our current study. We also found that leak respiration was significantly greater in intact platelet assay on an XFe96 versus O2K. Interestingly, the coupling control factor for oligomycin (i.e., 1–coupling control ratio for oligomycin) was markedly lower in intact platelets when assayed on an O2K versus an XFe96, meaning that calculated respiratory capacity available for ADP phosphorylation was far greater per platelet when assayed on an O2K versus an XFe96. These data suggest that caution should be made if comparing respiration data derived from XFe96 versus O2K experiments.

It is unclear why respiratory flux and coupling control data differ significantly across instrumental platforms. A potential factor driving this response may relate to cells being adhered to a microplate and respiration assayed in a small (~10 μL) unstirred volume in the XFe96 compared to respiration being assayed in cells suspended in a much larger (~2000 μL) volume of a stirred buffer in the O2K. Indeed, it is unclear whether plating or suspension in a vigorously stirred buffer differentially influences platelet aggregation and subsequently respiratory function. While in some cell types respiration in attached versus suspended cells is comparable (Zdrazilova et al., 2022), it is unclear if this is also true for platelets. One thing that was evident from our study was the variability within the data across the two platforms. For example, the coefficient of variance for three technical replicates assayed on different O2K instruments ranged from 1% to 16% (6% on average from *n* = 13 participant samples). Further, within a single O2K instrument, the coefficient of variance for technical replicates assayed in the two separate chambers of an O2K was 5% on average (from n = 13 participant samples). In contrast, the coefficient of variance for 5–6 technical replicates assayed on the XFe96 ranged from 8% to 57% (29% on average from *n* = 15 samples). Whether this variation arises from fundamental differences in the detection methods between these two platforms or results from the mechanical impact of adhering platelets to plastics is unclear, but suggests that respiratory function data derived from platelets adhered to plastic is more variable than that of data derived from platelets in suspension where respiration is assayed by an O_2_ electrode.

In summary, platelets represent a readily available sample to assay cellular bioenergetics, where a growing body of evidence suggests altered platelet bioenergetics in numerous physiological and pathophysiological settings. However, consideration should be given to sample preparation (i.e., intact versus permeabilized cells), assay buffer composition, and experimental protocol when planning experiments to leverage platelet respiratory function as a readout of cellular energetics. Further, caution should be exercised when comparing platelet respiratory function data derived from O2K and XFe96 platforms.

## Supporting information


**Table S1.** RPMI buffer composition.
**Table S2.** MIR05 buffer composition.
**Table S3.** Key supplies and chemicals used for blood collection, platelet isolation and respiration experiments.
**Table S4.** Buffer pH, osmolality, ionic strength and oxygen solubility.
**Table S5.** Respiration protocol 1 (XFe96).
**Table S6.** Respiration protocol 1 (O2K).
**Table S7.** Respiration protocol 2 (XFe96).
**Table S8.** Respiration protocol 2 (O2K).
**Table S9.** Respiration protocol 3 (O2K).
**Figure S1.** Respiratory responses to protonophore titration in permeabilized platelets assayed in either MIR05 or RPMI. State 3 and State 3U respiration (both supported by Complex I and II) from platelets from the same participants assayed in parallel in either MIR05 or RPMI are shown in Panels A and B, respectively. All measures of protonophore‐stimulated uncoupled respiration (state 3U) were made in the presence of oligomycin. Protonophore titration typically stimulated respiration in cells assayed in MIR05. In contrast, protonophore titration typically did not stimulate respiration in platelets assayed in RPMI. Panel C shows a trace of a parallel experiment in permeabilized platelets isolated from the same participant where platelets respond as anticipated to protonophore titration when assayed in MIR05, but failed to respond when assayed in RPMI. Individual values for each participant (*n* = 9) are plotted, as are group means ± standard deviations. *p < 0.05.
**Figure S2.** Coefficient of variance for routine respiration in intact platelets. Routine respiration assayed in intact platelets in RPMI containing 5 mM glucose, 1 mM pyruvate, and 2 mM glutamine. Individual values for each participant (*n* = 13) are plotted, as are the means ± standard deviations for these technical replicates. Individual measurements were made on a different O2K instrument albeit under identical assay conditions.
**Figure S3.** Relationship between platelet respiration and markers of cell mitochondrial protein levels. Panel A shows the relationship between cell protein content and citrate synthase (CS) activity. Panel B shows the relationship between routine respiration in intact platelets and cell protein concentration. Panel C shows the relationship between routine respiration in intact platelets and cell CS activity. Panel D shows the relationship between routine respiration in intact platelets and cell cytochrome C oxidase (COX) activity.
