# Supplementary material for: Mitochondrial respiratory function in human platelets: Influence of sample preparation, assay buffer, and instrumental platform
Source: Physiol Rep. 2025 Sep 30;13(19):e70555. doi: 10.14814/phy2.70555 (PMC12484291; doi:10.14814/phy2.70555)
Supplement: Supplementary file 1 — Table S1. RPMI buffer composition. Table S2. MIR05 buffer composition. Table S3. Key supplies and chemicals used for blood collection, platelet isolation and respiration experiments. Table S4. Buffer pH, osmolality, ionic strength and oxygen solubility. Table S5. Respiration protocol 1 (XFe96). Table S6. Respiration protocol 1 (O2K). Table S7. Respiration protocol 2 (XFe96). Table S8. Respiration protocol 2 (O2K). Table S9. Respiration protocol 3 (O2K). Figure S1. Respiratory responses to protonophore titration in permeabilized platelets assayed in either MIR05 or RPMI. State 3 and State 3U respiration (both supported by Complex I and II) from platelets from the same participants assayed in parallel in either MIR05 or RPMI are shown in Panels A and B, respectively. All measures of protonophore‐stimulated uncoupled respiration (state 3U) were made in the presence of oligomycin. Protonophore titration typically stimulated respiration in cells assayed in MIR05. In contrast, protonophore titration typically did not stimulate respiration in platelets assayed in RPMI. Panel C shows a trace of a parallel experiment in permeabilized platelets isolated from the same participant where platelets respond as anticipated to protonophore titration when assayed in MIR05, but failed to respond when assayed in RPMI. Individual values for each participant (n = 9) are plotted, as are group means ± standard deviations. *p < 0.05. Figure S2. Coefficient of variance for routine respiration in intact platelets. Routine respiration assayed in intact platelets in RPMI containing 5 mM glucose, 1 mM pyruvate, and 2 mM glutamine. Individual values for each participant (n = 13) are plotted, as are the means ± standard deviations for these technical replicates. Individual measurements were made on a different O2K instrument albeit under identical assay conditions. Figure S3. Relationship between platelet respiration and markers of cell mitochondrial protein levels. Panel A shows the relationship [file PHY2-13-e70555-s001.docx]

**Supplemental Materials**

**Supplemental Table** **1. RPMI buffer composition.**

| Solute | Final Concentration |
| --- | --- |
| Mg^2+^ (as MgSO_4_) | 0.4 mM |
| Ca^2+^ (as Ca(NO_3_)2•4H_2_O) | 0.4 mM |
| NaCl | 136.9 mM |
| KCl | 5.4 mM |
| Na_2_HPO_4_ | 5.6 mM |
| HEPES (N-2-hydroxyethylpiperazine-N-2-ethane sulfonic acid) | 1.0 mM |

**Supplemental Table 2. MIR05 buffer composition.**

| Solute | Final Concentration |
| --- | --- |
| EGTA | 0.5 mM |
| MgCl_2_.6 H_2_O | 3 mM |
| Lactobionic Acid | 60 mM |
| Taurine | 20 mM |
| KH_2_PO_4_ | 10 mM |
| D-Sucrose | 110 mM |
| HEPES (N-2-hydroxyethylpiperazine-N-2-ethane sulfonic acid) | 20 mM |
| BSA | 1% |

**Supplemental Table 3. Key supplies and chemicals used for blood collection, platelet isolation and respiration experiments.**

| **Chemical Name** | **Molecular weight** | **Vendor** | **Cat number** |
| --- | --- | --- | --- |
| 10x DPBS with Ca, Mg | n/a | Fisher | 14-080-055 |
| Vacutainers, K_2_EDTA, 6 mL, 100/pk | n/a | Fisher | 02-683-99D |
| Calibrant | n/a | Agilent | 103059-000 |
| RPMI | n/a | Agilent | 103681-100 |
| 1 M glucose solution | n/a | Agilent | 103577-100 |
| 100 mM pyruvate solution | n/a | Agilent | 103578-100 |
| 200 mM glucose solution | n/a | Agilent | 103579-100 |
| EGTA | 380.4 | Sigma | E4378 |
| MgCl_2_ ^.^6 H_2_O | 203.3 | Sigma | M0250 |
| Lactobionic acid | 358.3 | Sigma | 153516 |
| Taurine | 125.2 | Sigma | T0625 |
| KH_2_PO_4_ | 136.1 | Sigma | P5655 |
| HEPES | 238.3 | Sigma | H7523 |
| D-Sucrose | 342.3 | Sigma | S8501 |
| BSA, essentially fatty acid free | n/a | Boston BioProducts | P-753 |
| Digitonin | 1229.3 | Sigma | D5628 |
| Malate | 134.1 | Sigma | M1000 |
| Gutamate | 169.1 | Sigma | G1626 |
| ADP | 501.3 | Sigma | A5285 |
| Succinate | 270.1 | Sigma | S2378 |
| Oligomycin | 800 | Sigma | O4876 |
| FCCP | 254.2 | Sigma | C2920 |
| Rotenone | 394.4 | Sigma | R8875 |
| Antimycin A | 540 | Sigma | A8674 |
| Ascorbate | 198.1 | Sigma | A4034 |
| TMPD | 237.2 | Sigma | T3134 |

**Supplemental Table 4**. **Buffer pH, osmolality, ionic strength and oxygen solubility.**

|  | RMPI | MIR05 |
| --- | --- | --- |
| pH | 7.4 | 7.4 |
| Osmolality | 295 ± 15 mOsm | 330 mOsm |
| Ionic strength | ~150 mM | 95 mM |
| Solubility factor | 0.89 | 0.92 |

**Supplemental Table 5**. **Respiration protocol 1 (XFe96).**

| **Protocol 1 XFe96** | | | | |
| --- | --- | --- | --- | --- |
|  | **Inhibitor/Uncoupler** | **Stock Conc.** | **Addition** | **Final Conc.** |
| 1 | Oligomycin | 1mM | 1µM | 1µM |
| 2 | FCCP | 1mM | 1.5µM | 1.5µM |
| 3 | Rotenone | 1mM | 0.5µM | 0.5µM |
| 4 | Antimycin A | 5mM | 5µM | 5µM |

**Supplemental Table 6.** **Respiration protocol 1 (O2K).**

| **Protocol 1 O2K** | | | | |
| --- | --- | --- | --- | --- |
| **Step** | **Inhibitor/Uncoupler** | **Stock Conc.** | **Addition** | **Final Conc.** |
| 1 | Oligomycin | 1mM | 1µM | 1µM |
| 2 | FCCP | 1mM | 0.5µM | up to 1.5µM |
| 3 | Rotenone | 1mM | 0.5µM | 0.5µM |
| 4 | Antimycin A | 5mM | 5µM | 5µM |
| 5 | Ascorbate | 800mM | 2mM | 2mM |
|  | TMPD | 200mM | 0.5mM | 0.5mM |

**Supplemental Table 7. Respiration protocol 2 (XFe96).**

| **Protocol 2 XFe96** | | | | |
| --- | --- | --- | --- | --- |
| **Step** | **Inhibitor/Uncoupler** | **Stock Conc.** | **Addition** | **Final Conc.** |
| 1 | FCCP | 1mM | 1.5µM | 1.5µM |
| 2 | Rotenone | 1mM | 0.5µM | 0.5µM |
| 3 | Antimycin A | 5mM | 5µM | 5µM |

**Supplemental Table 8. Respiration protocol 2 (O2K).**

| **Protocol 2 O2K** | | | | |
| --- | --- | --- | --- | --- |
| **Step** | **Inhibitor/Uncoupler** | **Stock Conc.** | **Addition** | **Final Conc.** |
| 1 | FCCP | 1mM | 0.5µM | up to 1.5µM |
| 2 | Rotenone | 1mM | 0.5µM | 0.5µM |
| 3 | Antimycin A | 5mM | 5µM | 5µM |
| 4 | Ascorbate | 800mM | 2mM | 2mM |
|  | TMPD | 200mM | 0.5mM | 0.5mM |

**Supplemental Table 9. Respiration protocol 3 (O2K).**

| **Protocol 3 O2K** | | | | |
| --- | --- | --- | --- | --- |
| **Step** | **Inhibitor/Uncoupler** | **Stock Conc.** | **Addition** | **Final Conc.** |
| 1 | Digitonin | 8.1 mM | 8.1µM/10µg | 8.1µM |
| 2 | ADP | 500mM | 2.5mM | 2.5mM |
| 3 | Malate | 400mM | 2mM | 2mM |
|  | Glutamate | 2000mM | 10mM | 10mM |
| 4 | Succinate | 1000mM | 50mM | 50mM |
| 5 | Oligomycin | 1mM | 1µM | 1µM |
| 6 | FCCP | 1mM | 0.5µM | up to 1.5µM |
| 7 | Rotenone | 1mM | 0.5µM | 0.5µM |
| 8 | Antimycin A | 5mM | 5µM | 5µM |
| 9 | Ascorbate | 800mM | 2mM | 2mM |
|  | TMPD | 200mM | 0.5mM | 0.5mM |

**Supplemental Figure 1.**

**
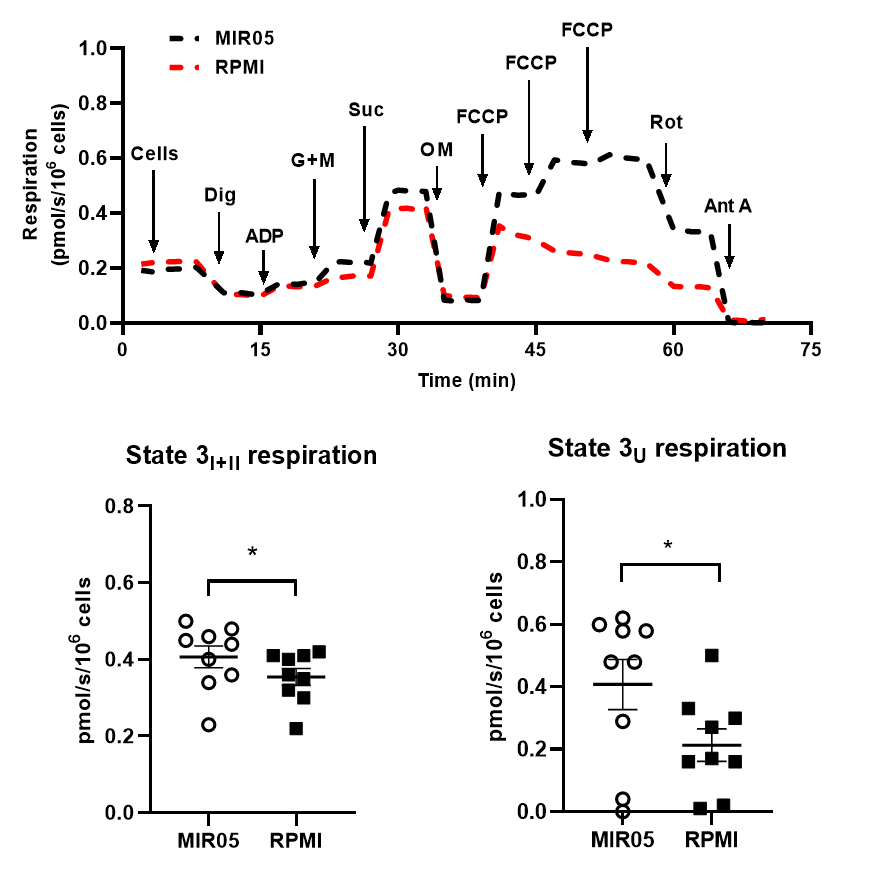
**

**Supplemental Figure 1. Respiratory responses to protonophore titration in permeabilized platelets assayed in either MIR05 or RPMI.** State 3 and State 3U respiration (both supported by Complex I and II) from platelets from the same participants assayed in parallel in either MIR05 or RPMI are shown in Panels A and B, respectively. All measures of protonophore-stimulated uncoupled respiration (state 3U) were made in the presence of oligomycin. Protonophore titration typically stimulated respiration in cells assayed in MIR05. In contrast, protonophore titration typically did not stimulate respiration in platelets assayed in RPMI. Panel C shows a trace of a parallel experiment in permeabilized platelets isolated from the same participant where platelets respond as anticipated to protonophore titration when assayed in MIR05, but failed to respond when assayed in RPMI. Individual values for each participant (n = 9) are plotted, as are group means ± standard deviations. *P<0.05.

**Supplemental Figure 2.**

**Supplemental Figure 2. Coefficient of variance for routine respiration in intact platelets.** Routine respiration assayed in intact platelets in RPMI containing 5 mM glucose, 1 mM pyruvate, and 2 mM glutamine. Individual values for each participant (n=13) are plotted, as are the means ± standard deviations for these technical replicates. Individual measurements were made on a different O2K instrument albeit under identical assay conditions.

**Supplemental Figure 3.**


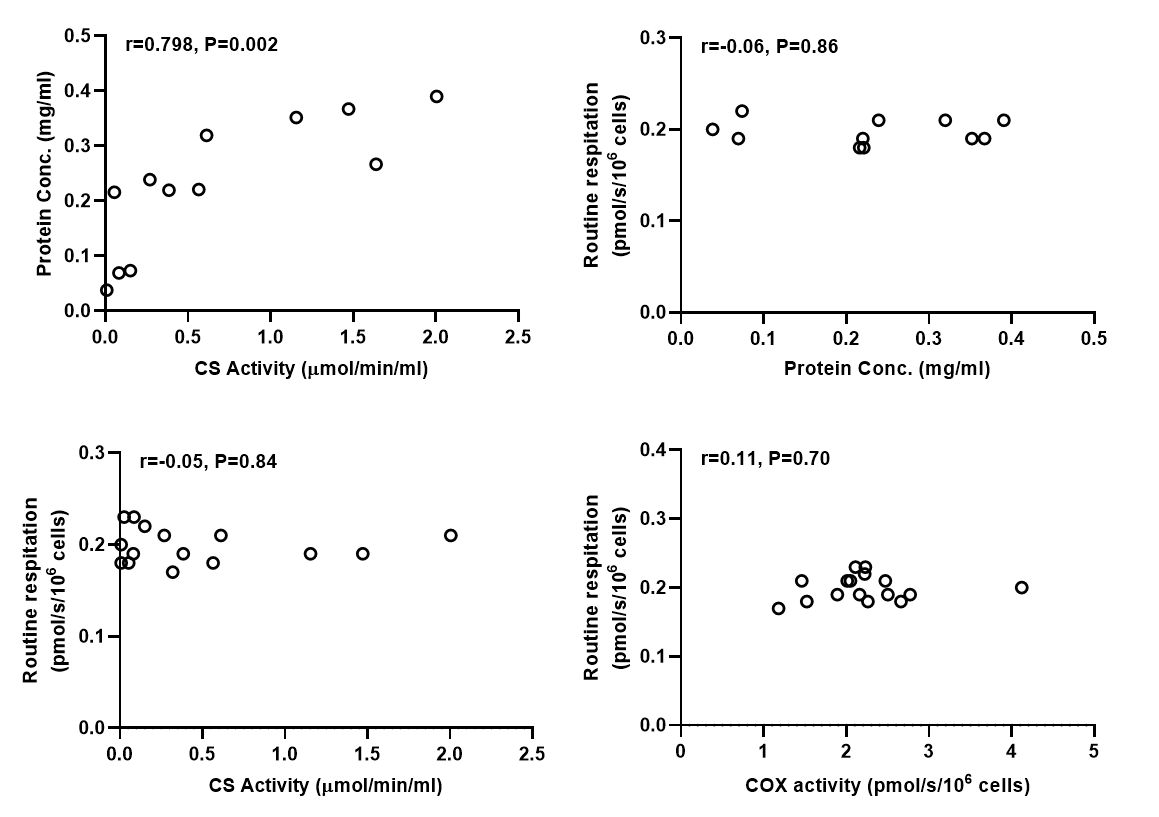


**Supplemental Figure 3. Relationship between platelet respiration and markers of cell mitochondrial protein levels.** Panel A shows the relationship between cell protein content and citrate synthase (CS) activity. Panel B shows the relationship between routine respiration in intact platelets and cell protein concentration. Panel C shows the relationship between routine respiration in intact platelets and cell CS activity. Panel D shows the relationship between routine respiration in intact platelets and cell cytochrome C oxidase (COX) activity.
